# Letter from the Editor in Chief

**DOI:** 10.19102/icrm.2026.17036

**Published:** 2026-03-15

**Authors:** Devi Nair


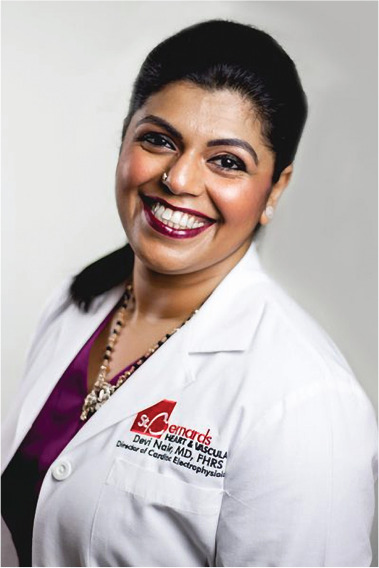


Dear Colleagues,

Welcome to the March 2026 issue of *Innovations in Cardiac Rhythm Management*.

March is always a dynamic moment in our field, bridging two highly anticipated scientific gatherings. The Western AF Symposium in Park City continues to distinguish itself as a uniquely interactive and forward-looking forum, emphasizing practical strategy, operator experience, and nuanced decision-making in atrial fibrillation care. This year’s sessions reinforced themes that are increasingly central to modern electrophysiology: durability of ablation, individualized rhythm-control strategies, and the integration of pharmacologic and procedural approaches.

Closely following this, the upcoming American College of Cardiology (ACC) Scientific Sessions promises to broaden the conversation further, bringing together late-breaking clinical trials, guideline updates, and multidisciplinary perspectives that will shape cardiovascular care at large. Increasingly, such sessions are also highlighting the rapid emergence of artificial intelligence (AI) in cardiac electrophysiology, from AI-enabled mapping systems and signal interpretation to imaging integration and workflow optimization. The ability to synthesize large-scale electroanatomic, imaging, and clinical datasets in real time is beginning to influence how we approach atrial fibrillation and ventricular arrhythmia ablation, refine patient selection, and even predict outcomes. Importantly, the conversation is shifting from feasibility to implementation, with an exploration of how these tools can be validated, standardized, and meaningfully integrated into daily practice.

Together, these meetings reflect a field that is not only innovating rapidly but also critically evaluating how best to translate innovations, including digital and AI-driven technologies, into durable patient outcomes.

The manuscripts in this issue mirror many of these themes, particularly the ongoing tension and opportunity between pharmacologic and interventional rhythm control.

In this month’s original investigation, Ardıç et al.^1^ explore an important and common clinical dilemma: how best to manage recurrent atrial fibrillation following cryoballoon ablation. Their study demonstrates that flecainide achieves rhythm control comparable to repeat radiofrequency ablation, with significantly better outcomes than propafenone. Notably, left atrial size emerged as a key determinant of recurrence, reinforcing the importance of substrate in guiding therapy selection. These findings resonate strongly with current discussions in the field, particularly those highlighted at Western AF, around tailoring therapy based not only on prior procedural history but also on underlying atrial remodeling and, increasingly, on data-driven risk-stratification approaches.

Device innovation is another prominent theme in this issue. Yokokawa et al.^2^ present a compelling case of leadless pacemaker implantation during pregnancy, demonstrating the feasibility of achieving effective pacing with minimal fluoroscopy exposure. This report underscores how technological advances can expand treatment options in traditionally high-risk scenarios, emphasizing safety without compromising efficacy. As device therapy continues to evolve, and as imaging and AI-guided implantation strategies mature, such cases highlight the importance of thoughtful patient selection and multidisciplinary collaboration.

In the realm of ventricular arrhythmias, Ozeke et al.^3^ provide a striking example of ventricular tachycardia termination via prepotential-to–myocardial exit block. The case elegantly illustrates fundamental electrophysiologic principles, particularly the role of discrete prepotentials and conduction delay in arrhythmia manifestation and ablation targeting. Even as mapping technologies become increasingly sophisticated with growing incorporation of automated annotation and AI-assisted interpretation, these mechanistic insights remain essential to successful intervention.

Taken together, the contributions in this issue highlight a central theme echoed across recent and upcoming meetings: precision in electrophysiology. Whether selecting anti-arrhythmic therapy after failed ablation, adapting device strategies to complex physiologic states, or leveraging emerging digital and AI-enabled tools to guide intervention, the field continues to move toward increasingly individualized care.

As we look ahead to the ACC Scientific Sessions, we anticipate further clarity on many of these questions, particularly regarding long-term outcomes, comparative effectiveness, and the responsible integration of AI and advanced technologies into everyday clinical practice.

I extend my sincere thanks to our authors and reviewers for their contributions, and to our readers for their continued engagement in advancing the science and practice of cardiac electrophysiology.

Warm regards,


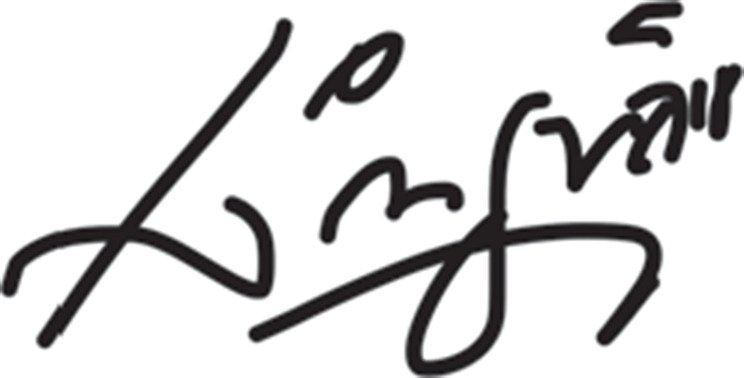


Dr. Devi Nair, md, facc, fhrs

Editor-in-Chief

*The Journal of Innovations in Cardiac Rhythm Management*

Director of the Cardiac Electrophysiology & Research,

St. Bernard’s Heart & Vascular Center, Jonesboro, AR, USA

White River Medical Center, Batesville, AR, USA

President/CEO, Arrhythmia Research Group

Clinical Adjunct Professor, University of Arkansas for Medical Sciences

Governor, Arkansas Chapter of the American College of Cardiology

drdgnair@gmail.com
